# Association between calf circumference and cardiovascular health indicators in patients with peripheral artery disease

**DOI:** 10.31744/einstein_journal/2025AO1089

**Published:** 2025-08-15

**Authors:** Fabiana Gonçalves Ferreira, Hélcio Kanegusuku, Wellington Segheto, Gabriel Grizzo Cucato, Nelson Wolosker, Raphael Mendes Ritti-Dias, Rosilene Motta Elias, Breno Quintella Farah, Marilia de Almeida Correia

**Affiliations:** 1 Universidade Nove de Julho Postgraduate Program in Medicine São Paulo SP Brazil Postgraduate Program in Medicine, Universidade Nove de Julho, São Paulo, SP, Brazil.; 2 Hospital Israelita Albert Einstein São Paulo SP Brazil Hospital Israelita Albert Einstein São Paulo, SP, Brazil.; 3 Universidade Federal de Lavras Graduate Program in Health and Nutrition Lavras MG Brazil Graduate Program in Health and Nutrition, Universidade Federal de Lavras, Lavras, MG, Brazil.; 4 Northumbria University Department of Sport, Exercise and Rehabilitation England Department of Sport, Exercise and Rehabilitation, Northumbria University, England.; 5 Universidade de São Paulo Faculdade de Medicina São Paulo SP Brazil Faculdade de Medicina, Universidade de São Paulo, São Paulo, SP, Brazil.; 6 Hospital Israelita Albert Einstein Hospital Municipal Gilson de Cássia Marques de Carvalho São Paulo SP Brazil Hospital Municipal Gilson de Cássia Marques de Carvalho; Hospital Israelita Albert Einstein, São Paulo, SP, Brazil.; 7 Universidade Nove de Julho Postgraduate Program in Rehabilitation Sciences São Paulo SP Brazil Postgraduate Program in Rehabilitation Sciences, Universidade Nove de Julho, São Paulo, SP, Brazil.; 8 Universidade Federal Rural de Pernambuco Department of Physical Education Recife PE Brazil Department of Physical Education, Universidade Federal Rural de Pernambuco, Recife, PE, Brazil.

**Keywords:** Peripheral arterial disease, Cardiovascular diseases, Anthropometry, Calf circumference, Vascular stiffness, Blood pressure, Carotid-femoral pulse wave velocity, Ankle brachial index, Leg

## Abstract

**Objective:**

To analyze the association between calf circumference and cardiovascular indicators in men and women with symptomatic peripheral artery disease.

**Methods:**

This cross-sectional study included 259 patients with peripheral artery disease (65% men; mean age, 67 [±12] years; ankle brachial index, 0.58 [0.26]). We measured calf circumference, brachial and central blood pressure, arterial stiffness indicators (carotid-femoral pulse wave velocity and augmentation index), and heart rate variability parameters. Multiple linear regression was used to evaluate the association between calf circumference and cardiovascular indicators after adjusting for age, ankle-brachial index, and walking capacity.

**Results:**

In women, calf circumference was inversely associated with central blood pressure (systolic: β=-1.91 [0.70] mmHg, p=0.008; diastolic: β=-0.97 [0.29] mmHg, p=0.001), augmentation index (β=-1.06 [0.28]%, p<0.001), and augmentation index corrected by 75 bpm (β=-0.90 [0.23]%, p<0.001), independent of age, ankle brachial index, and walking capacity. After further adjustments for anti-hypertensive medication use and body mass index, carotid-femoral pulse wave velocity was the only parameter associated with calf circumference (β=-0.41 [0.15] m/s, p=0.010). In men, no significant association was observed between calf circumference and cardiovascular indicators (p>0.05).

**Conclusion:**

In women with peripheral artery disease, calf circumference was inversely associated with arterial stiffness. This finding suggests that a lower calf circumference may serve as an indicator of increased arterial stiffness in women with peripheral artery disease.

## In Brief

This study aimed to analyze the association between calf circumference and cardiovascular indicators in patients with peripheral artery disease. In women, calf circumference was inversely associated with arterial stiffness, suggesting that a lower calf circumference may indicate increased arterial stiffness. No significant associations were observed among men.

## Highlights

■ Calf circumference is a well-established indicator of all-cause mortality.
■ In women with peripheral artery disease, calf circumference was inversely associated with arterial stiffness.
■ Among men with peripheral artery disease, calf circumference was not associated with cardiovascular indicators.
■ Calf circumference may be a useful screening tool for cardiovascular risk in women.

## Graphical abstract

**Figure:**
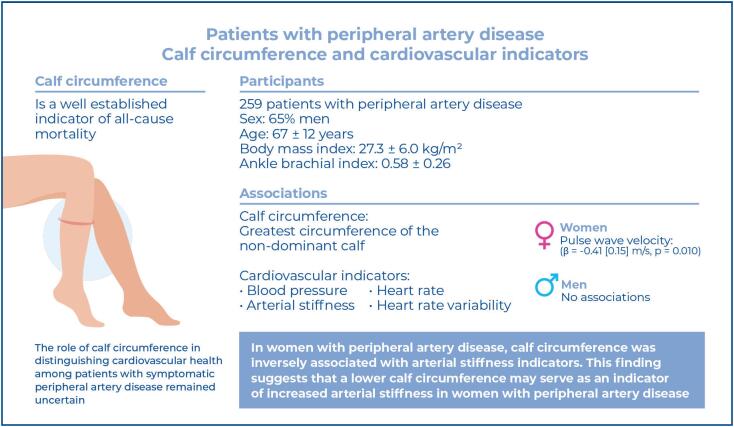

## INTRODUCTION

Peripheral artery disease (PAD) is more common in older adults^(1)^ and affects the structure and function of arteries in the lower extremities, thereby reducing the oxygen supply to peripheral tissues.^(2)^ The primary symptom reported by patients with PAD is intermittent claudication.^(2)^ Patients with symptomatic PAD typically experience reduced muscle mass^(3,4)^ and strength,^(5)^ as well as mitochondrial dysfunction^(4,6)^ in the lower limbs. Additionally, patients with PAD exhibit cardiovascular issues, such as high blood pressure,^(7)^ arterial stiffness,^(8)^ and autonomic dysfunction.^(9)^ These factors contribute, in part, to an increased risk of cardiovascular morbidity and mortality.^(10,11)^

Calf circumference is considered a significant nutritional indicator in older adults^(12)^ and has implications for cardiovascular mortality. Data from the National Health and Nutrition Examination Survey have revealed an association between lower calf circumference and an increased risk of cardiovascular and all-cause mortality.^(13)^ However, whether calf circumference is an indicator of cardiovascular health in patients with symptomatic PAD remains unknown.

## OBJECTIVE

This study aimed to analyze the association between calf circumference and cardiovascular indicators in men and women with symptomatic peripheral artery disease.

## METHODS

### Study design and ethical considerations

This cross-sectional study followed the Strengthening the Reporting of Observational Studies in Epidemiology (STROBE) checklist. This study was approved by the Research Ethics Committees of *Hospital Israelita Albert Einstein*, CAAE: 42379015.3.0000.0071; #4,435,316 and *Hospital das Clínicas, Faculdade de Medicina, Universidade de São Paulo*, CAAE: 42379015.3.3002.0068, # 4.614.795. Each patient was informed about the risks and benefits of the study and provided written informed consent for participation. The procedures used in this study have been previously described.^(7,14)^

### Recruitment and patients

Patients were recruited after routine appointments at the Vascular Units at the *Hospital Municipal Gilson de Cássia Marques de Carvalho* of the *Hospital Israelita Albert Einstein* and the *Hospital das Clínicas* of the *Universidade de São Paulo*, Brazil, between September 2015 and November 2019. The inclusion criteria were: a) age ≥50 years; b) ankle brachial index ≤0.90 in one or both legs; c) absence of non-compressible vessels, amputated limbs, or ulcers; and d) absence of unstable cardiac diseaseThe total sample size comprised 259 patients.

### Outcomes

#### Blood pressure

Brachial blood pressure (BP) was assessed in the supine position using an automatic monitor (HEM 742; Omron Healthcare, Japan), as previously described.^(7)^ Three consecutive measurements were performed, with a 1-min interval between measurements, in both arms, and with the appropriate cuff size. The value used was the highest of the two arms.^(15)^

Central BP was obtained through the pulse wave analysis recorded in the radial artery using applanation tonometry (SphygmoCor AtcorMedical, Sydney, Australia). A validated transfer function algorithm, provided by the Sphygmocor^®^ software, was used to estimate the central values of systolic and diastolic BP.^(16)^

#### Arterial stiffness

The augmentation index (proportion of pulse pressure attributed to the reflected pulse wave) was obtained using applanation tonometry (SphygmoCor; AtCor Medical, Australia) in the radial artery, as previously described. Carotid-femoral pulse wave velocity was measured using applanation tonometry (Sphygmocor; AtCor Medical, Australia) following the guidelines of the Clinical Application of Arterial Stiffness Task Force III.^(17)^ For carotid-femoral pulse wave velocity measurement, the distances from the carotid artery to the suprasternal notch and from the femoral artery to the suprasternal notch were measured using standard tape. A simultaneous electrocardiogram was recorded to obtain time measurements through heart rate. Using the "foot-to-foot" method, the time difference between the points was measured. Subsequently, the distance between the two arteries was divided according to the time difference.

#### Cardiac autonomic modulation

Cardiac autonomic modulation was assessed through heart rate variability, from records of the beat-to-beat intervals obtained using an HR monitor (RS800CX, Polar Electro, Finland) during 10 min. All analyses were performed using Kubios HRV software (version 3.5.0, Biosignal Analysis and Medical Imaging Group, Kuopio, Finland) following the recommendations of the Task Force for HRV.^(18)^ The time-domain parameters measured were standard deviation of all RR intervals (SDNN), root mean square of the squared differences between adjacent normal RR intervals (RMSSD), and the percentage of adjacent intervals over 50 ms (pNN50). SDDN was used as a marker of total variability (cardiac sympathetic and parasympathetic modulations), and RMSSD and pNN50 as markers of predominant cardiac parasympathetic modulations. In addition, the frequency-domain variables were calculated using Fast Fourier Transform. The spectral components were assigned based on their central frequency, as low (0.04–0.15 Hz) and high frequency (0.15–0.4 Hz). Low- and high-frequency components were accepted as markers of predominant cardiac sympathetic and parasympathetic modulation, respectively, whereas the low-frequency/high-frequency ratio was considered as the cardiac sympathovagal balance.

#### Calf circumference predictors

Calf circumference was measured at the greatest circumference of the nondominant calf using an inelastic measuring tape with an accuracy of 0.01cm.

#### Confounders

Demographic data (sex and age), ankle-brachial index, and walking capacity were assessed. The ankle-brachial index was evaluated according to a previously described protocol.^(19)^ Walking capacity was assessed using the 6-min walk test, which was performed along a 30-m-long corridor, as previously described.^(20)^ Briefly, the patients were encouraged to walk at their usual pace for 6 min and cover as much distance as possible, resting if necessary. The total walking distance was defined as the maximum distance reached by the patient at the end of the test. Claudication onset distance was defined as the distance walked until the patient first reported leg pain.

### Statistical analysis

Statistical analyses were performed using SPSS version 20 software (IBM Corp., Armonk, NY, USA). Data normality was analyzed using the Kolmogorov–Smirnov test. Continuous data are presented as median and interquartile range, and categorical data are presented as frequency. Comparisons between men and women were performed using the Mann–Whitney U test (continuous variables) and Pearson's chi-square test (categorical variables).

The association between calf circumference and cardiovascular indicators was analyzed using multiple linear regression. All analyses were stratified by sex and adjusted for age, ankle-brachial index, and 6-min walk test (Model 1) and for Model 1 plus hypertension medication and body mass index (Model 2). Residual analysis was performed using graphical analysis (histograms). Statistical significance was set at p<0.050.

## RESULTS

The clinical characteristics of the patients are presented in table 1. Men had a higher calf circumference, claudication onset distance, and 6-min walk distance, and a lower prevalence of dyslipidemia and use of angiotensin receptor agonists than women. Other clinical characteristics were similar between the sexes.

**Table 1:** Characteristics of patients with peripheral artery disease

Variables		n	All (n=259)	Men (n=169)	Women (n=90)	p value
Age, years old		259	67 (12)	67 (12)	67 (11)	0.989
Body mass index, kg/m^2^		257	27.3 (6.0)	27.3 (5.7)	27.3 (6.8)	0.488
Ankle brachial index		259	0.58 (0.26)	0.57 (0.25)	0.58 (0.23)	0.427
Calf circumference, cm		259	35.0 (5.0)	35.0 (4.6)	34.0 (4.1)	0.001
Claudication onset distance, m		198	117 (99)	120 (94)	96 (11)	0.001
6-min walk distance, m		198	327 (117)	335 (117)	300 (116)	0.002
Relative 6-min walk distance, %		223	60.8 (20.8)	61.0 (19.9)	58.9 (21.9)	0.742
Weight-walking distance product, km.kg		223	22.7 (10.4)	25.1 (10.0)	20.5 (9.5)	<0.001
Risk factors, %						
	Diabetes	252	52	50	56	0.381
	Dyslipidemia	248	80	76	89	0.015
	Coronary artery disease	244	33	33	34	0.899
	Hypertension	251	84	83	87	0.354
Medication, %						
	ACEi	204	27	28	24	0.494
	Antiplatelet	204	86	86	85	0.748
	ARA	204	30	24	42	0.010
	Beta-blockers	204	41	38	47	0.195
	Diuretics	204	42	42	42	0.917
	Hypoglycemics	229	44	43	46	0.564
	Statins	204	89	88	90	0.605
	Vasodilators	204	28	31	24	0.260

Data are presented as median (interquartile range) or relative frequency.

ACEi: angiotensin-converting enzyme inhibitor; ARA: angiotensin receptor antagonist.

The cardiovascular indicators are shown in table 2. Men had lower brachial systolic BP than women. Other cardiovascular indicators were similar between the sexes.

**Table 2:** Cardiovascular indicators of patients with peripheral artery disease

Variables		n	All	Men	Women	p value
Blood pressure, mmHg						
	Brachial systolic BP	259	137 (31)	134 (28)	142 (28)	0.005
	Brachial diastolic BP	259	72 (14)	73 (14)	72 (16)	0.600
	Central systolic BP	227	130 (34)	128 (30)	133 (34)	0.241
	Central diastolic BP	227	76 (13)	75 (14)	77 (15)	0.208
Arterial stiffness						
	Pulse pressure, mmHg	227	55 (28)	55 (28)	56 (27)	0.494
	Augmentation index, %	226	32 (14)	31 (14)	34 (16)	0.193
	Augmentation index 75 bpm, %	226	28 (11)	28 (12)	28 (11)	0.692
	Pulse wave velocity, m/s	176	9.2 (4.0)	9.1 (4.0)	9.2 (4.3)	0.609
Cardiac autonomic modulation						
	SDNN, ms	204	32 (41)	31 (43)	33 (33)	0.794
	RMSSD, ms	204	27 (41)	27 (43)	27 (32)	0.725
	pNN50, %	204	3 (17)	2.9 (17)	4.6 (21)	0.345
	Low frequency, nu	204	51 (34)	51 (34)	53 (33)	0.555
	High frequency, nu	204	48 (34)	49 (33)	46 (34)	0.544
	Low frequency/high frequency	204	1.0 (1)	1.0 (1.6)	1.1 (2.1)	0.380

Data are presented as median (interquartile range).

BP: blood pressure; SDNN: standard deviation of all RR intervals; RMSSD: root mean square of the squared differences between adjacent normal RR intervals; pNN50: percentage of adjacent intervals >50ms.

Table 3 presents the results of the association analysis between calf circumference and cardiovascular indicators in women. Calf circumference was inversely associated (p<0.050) with central systolic and diastolic BP (β=-1.91 [0.70] mmHg, p=0.008 and β=-0.97 [0.29] mmHg, p=0.001, respectively, augmentation index (β=-1.06 [0.28]%, p<0.001), and augmentation index corrected by 75bpm (β=-0.90 [0.23]%, p<0.001), independent of age, ankle brachial index, and walking capacity. After further adjustments for anti-hypertensive medication use and body mass index, carotid-femoral pulse wave velocity was the only parameter associated with calf circumference (β=-0.41 [0.15] m/s, p=0.010). In addition, heart rate variability was not significantly associated with calf circumference (p>0.050).

**Table 3:** Multiple linear regression analyses of the associations between calf circumference and cardiovascular indicators in women with peripheral artery disease

Outcomes		Model 1				Model 2			
		β (SE)	b	p value	F (p value)*	β (SE)	b	p value	F (p value)*
Blood pressure, mmHg									
	Brachial systolic BP	-1.12 (0.56)	-0.239	0.048	2.083 (0.092)	-0.46 (0.80)	-0.110	0.567	0.842 (0.543)
	Brachial diastolic BP	-0.43 (0.27)	-0.188	0.122	1.570 (0.191)	-0.51 (0.36)	-0.265	0.155	1.642 (0.153)
	Central systolic BP	-1.91 (0.70)	-0.326	0.008	3.305 (0.016)	-0.65 (1.24)	-0.106	0.602	1.953 (0.091)
	Central diastolic BP	-0.97 (0.29)	-0.392	0.001	3.943 (0.006)	-1.16 (0.52)	-0.452	0.030	1.856 (0.108)
Arterial stiffness									
	Augmentation index, %	-1.06 (0.28)	-0.430	<0.001	4.549 (0.003)	-0.60 (0.47)	-0.241	0.203	3.535 (0.006)
	Augmentation index 75 bpm, %	-0.90 (0.23)	-0.458	<0.001	4.048 (0.005)	-0.87 (0.39)	-0.447	0.030	2.113 (0.068)
	Pulse wave velocity, m/s	-0.14 (0.11)	-0.195	0.209	1.459 (0.229)	-0.41 (0.15)	-0.576	0.010	3.256 (0.011)
Cardiac autonomic modulation									
	SDNN, ms	-0.23 (2.03)	-0.015	0.911	0.831 (0.512)	-1.19 (1.83)	-0.119	0.519	0.890 (0.511)
	RMSSD, ms	-0.88 (2.38)	-0.049	0.714	1.301 (0.281)	-2.23 (2.54)	-0.156	0.385	1.292 (0.281)
	pNN50, %	0.68 (0.70)	0.130	0.337	0.940 (0.448)	0.98 (0.96)	0.190	0.313	0.570 (0.752)
	Low frequency, nu	-1.03 (0.76)	-0.177	0.184	1.587 (0.191)	-1.16 (1.09)	-0.193	0.291	1.030 (0.419)
	High frequency, nu	1.04 (0.76)	0.180	0.176	1.585 (0.191)	1.18 (1.09)	0.197	0.281	1.030 (0.419)
	Low frequency/high frequency ratio	-0.07 (0.05)	-0.179	0.174	1.927 (0.119)	-0.08 (0.08)	-0.196	0.282	1.080 (0.389)

* Model 1 was adjusted for age, 6-min walk distance, and ankle-brachial index. Model 2 was adjusted for Model 1 plus hypertension medication and body mass index.

BP: blood pressure; SDNN: standard deviation of all RR intervals; RMSSD: root mean square of the squared differences between adjacent normal RR intervals; pNN50: percentage of adjacent intervals >50ms.

Table 4 shows the results of the association analysis between calf circumference and cardiovascular indicators in men. No significant association was observed between calf circumference and cardiovascular indicators in men (p>0.05).

**Table 4:** Multiple linear regression analyses of the associations between calf circumference and cardiovascular indicators in men with peripheral artery disease

Outcomes		Model 1				Model 2			
		β (SE)	b	p value	F (p value)*	β (SE)	b	p value	F (p value)*
Blood pressure, mmHg									
	Brachial systolic BP	-0.22 (0.44)	-0.040	0.623	3.201 (0.015)	0.18 (0.75)	0.031	0.806	2.057 (0.064)
	Brachial diastolic BP	0.17 (0.22)	0.064	0.438	2.441 (0.050)	0.65 (0.34)	0.235	0.060	2.496 (0.027)
	Central systolic BP	0.86 (0.50)	-0.154	0.086	1.941 (0.108)	0.09 (0.88)	0.015	0.918	1.964 (0.079)
	Central diastolic BP	-0.05 (0.22)	-0.021	0.814	1.322 (0.262)	0.51 (0.38)	0.193	0.189	1.119 (0.358)
Arterial stiffness									
	Augmentation index, %	-0.14 (0.23)	-0.054	0.546	1.989 (0.100)	-0.16 (0.39)	-0.059	0.676	2.031 (0.070)
	Augmentation index 75bpm, %	-0.29 (0.21)	-0.121	0.175	1.900 (0.115)	-0.38 (0.37)	-0.142	0.318	2.124 (0.058)
	Pulse wave velocity, m/s	-0.04 (0.07)	-0.057	0.570	2.207 (0.074)	-0.06 (0.10)	-0.086	0.545	4.951 (<0.001)
Cardiac autonomic modulation									
	SDNN, ms	1.77 (1.02)	0.163	0.085	1.644 (0.168)	3.03 (1.75)	0.236	0.088	1.872 (0.095)
	RMSSD, ms	2.21 (1.44)	0.144	0.128	1.727 (0.149)	3.60 (2.47)	0.200	0.148	1.817 (0.105)
	pNN50, %	0.64 (0.38)	0.155	0.100	2.011 (0.098)	1.20 (0.62)	0.266	0.056	1.699 (0.131)
	Low frequency, nu	-0.82 (0.48)	-0.160	0.087	2.606 (0.040)	-0.01 (0.79)	-0.001	0.994	2.495 (0.028)
	High frequency, nu	0.82 (0.48)	0.159	0.089	2.622 (0.039)	0.01 (0.78)	0.001	0.993	2.495 (0.028)
	Low frequency/high frequency ratio	-0.03 (0.07)	-0.041	0.666	0.930 (0.450)	0.20 (0.13)	0.205	0.142	1.545 (0.173)

* Model 1 was adjusted for age, 6-min walk distance, and ankle-brachial index. Model 2 was adjusted for Model 1 plus hypertension medication and body mass index.

BP: blood pressure; SDNN: standard deviation of all RR intervals; RMSSD: root mean square of the squared differences between adjacent normal RR intervals; pNN50: percentage of adjacent intervals >50ms.

## DISCUSSION

The main findings of this study were as follows: (i) in women with PAD, calf circumference, a marker of skeletal muscle mass, was significantly associated with central BP and arterial stiffness indicators, augmentation index, and augmentation index corrected to 75 bpm, after adjusting for age, ankle brachial index, and 6-min walk distance; (ii) after further adjusting for medication use and body mass index, only carotid-femoral pulse wave velocity was significantly associated with calf circumference in women; and (iii) in men, calf circumference was not associated with any of the evaluated cardiovascular parameters.

To our knowledge, this is the first study to investigate the relationship between cardiovascular parameters and calf circumference in patients with PAD, which limits comparison with existing literature. Patients with PAD are known to present with impairments in cardiac autonomic control, increased arterial stiffness, and altered BP, thereby necessitating frequent use of medication for management. In these patients, the calf muscle exhibits atrophy and altered fiber proportions, with increased type I fibers compared to age-matched controls.^(21)^ Comparisons with other populations are difficult because of the unique impact of PAD on cardiovascular function and calf muscles.

Our study revealed that calf circumference was inversely associated with central BP but not with brachial BP. Additionally, we observed an inverse association between the calf circumference and wave reflection indicators (augmentation index and augmentation index corrected to 75bpm). These associations disappeared after adjusting for medication use and body mass index, suggesting a possible direct influence of medication and body mass index on the central BP and wave reflection parameters in patients with PAD.

Our study's main finding was the relationship between skeletal muscle mass, assessed using calf circumference, and arterial stiffness, measured using carotid-femoral pulse wave velocity, the gold standard clinical marker of arterial stiffness. A previous meta-analysis, including cross-sectional studies, showed a negative correlation between muscle mass and peak wave velocity.^(22)^ Arterial stiffness is directly related to blood supply to tissues. The most plausible mechanism explaining this relationship is that increased arterial stiffness can reduce blood flow to the muscles, impairing metabolism and, consequently, muscle composition.^(23)^ A less likely hypothesis is that a decreased muscle mass causes greater arterial stiffness. Muscle atrophy may lead to the release of damaging cytokines and oxidative stress, which, in turn, can impair glucose metabolism and disrupt hormone regulation,^(24)^ ultimately increasing stiffness. Future studies should explore the mechanistic pathways involved in the relationship between muscle mass and arterial stiffness in patients with PAD.

The fact that the association between calf circumference and arterial stiffness only occurred in women is notable. Previous studies suggest that women with PAD typically exhibit poorer physical fitness in the lower limbs, lower muscle mass, and worse cardiovascular function than men.^(25,26)^ Additionally, Gardner et al.^(27)^ showed that women reach minimum calf muscle StO_2_ within a shorter time during treadmill exercise, indicating a greater impairment in the increase in capillary blood volume during exercise. This finding supports the idea that arterial stiffness may be related to muscle impairments observed in women.

Regarding heart rate variability, patients with PAD generally exhibit increased cardiac sympathetic modulation.^(9)^ Abreo et al.^(28)^ observed a significant association between higher thigh circumference and a lower risk of cardiovascular mortality in individuals without PAD aged over 20 years. However, in the present study, calf circumference was not significantly associated with any of the parameters evaluated. Notably, these associations lost significance after adjusting for age, ankle-brachial index, and walking capacity, suggesting that these factors may be more reliable indicators of autonomic dysfunction than calf circumference in patients with PAD.

The key strengths of our study include the robust sample size, the use of high-quality methods to assess cardiovascular indicators, and the blinding of evaluators responsible for the cardiovascular measurements to the calf circumference. However, this study had certain limitations. The cross-sectional design prevented the establishment of causal relationships; thus, longitudinal studies should be conducted to better understand the effect of calf circumference in patients with PAD. In addition, patients used various medications to control risk factors, such as diabetes, dyslipidemia, and hypertension, which are the main confounders when analyzing cardiovascular indicators. However, this increases the external validity of the results.

## CONCLUSION

Calf circumference is inversely associated with arterial stiffness indicators in women with peripheral artery disease. This finding suggests that lower calf circumference may serve as an indicator of increased arterial stiffness in women with peripheral artery disease. Future studies should investigate the mechanisms underlying this relationship.

## References

[1] GBD 2019 Peripheral Artery Disease Collaborators (2023). Global burden of peripheral artery disease and its risk factors, 1990-2019: a systematic analysis for the Global Burden of Disease Study 2019. Lancet Glob Health.

[2] Fowkes FG, Aboyans V, Fowkes FJ, McDermott MM, Sampson UK, Criqui MH (2017). Peripheral artery disease: epidemiology and global perspectives. Nat Rev Cardiol.

[3] Nishibe T, Kano M, Akiyama S, Chiba F, Nishibe M, Koizumi J (2023). Influence of Lower Limb Ischemia on Skeletal Muscle Mass Depletion in Patients with Peripheral Artery Disease. Ann Vasc Surg.

[4] Pizzimenti M, Meyer A, Charles AL, Giannini M, Chakfé N, Lejay A (2020). Sarcopenia and peripheral arterial disease: a systematic review. J Cachexia Sarcopenia Muscle.

[5] Câmara LC, Ritti-Dias RM, Menêses AL, D'Andréa Greve JM, Filho WJ, Santarém JM (2012). Isokinetic strength and endurance in proximal and distal muscles in patients with peripheral artery disease. Ann Vasc Surg.

[6] Park SY, Pekas EJ, Anderson CP, Kambis TN, Mishra PK, Schieber MN (2022). Impaired microcirculatory function, mitochondrial respiration, and oxygen utilization in skeletal muscle of claudicating patients with peripheral artery disease. Am J Physiol Heart Circ Physiol.

[7] Farah BQ, Cucato GG, Andrade-Lima A, Soares AH, Wolosker N, Ritti-Dias RM (2021). Impact of hypertension on arterial stiffness and cardiac autonomic modulation in patients with peripheral artery disease: a cross-sectional study. einstein (São Paulo).

[8] Zahner GJ, Gruendl MA, Spaulding KA, Schaller MS, Hills NK, Gasper WJ (2017). Association between arterial stiffness and peripheral artery disease as measured by radial artery tonometry. J Vasc Surg.

[9] Cachadiña ES, De la Cruz Torres B, Sixto AS, Martín PF, Berral de la Rosa FJ, Orellana JN (2018). Heart rate variability is lower in patients with intermittent claudication: A preliminary study. Arch Med Deporte.

[10] Aursulesei Onofrei V, Ceasovschih A, Marcu DT, Adam CA, Mitu O, Mitu F (2022). Mortality Risk Assessment in Peripheral Arterial Disease-The Burden of Cardiovascular Risk Factors over the Years: A Single Center's Experience. Diagnostics (Basel).

[11] Scandale G, Dimitrov G, Recchia M, Carzaniga G, Perilli E, Carotta M (2020). Arterial stiffness and 5-year mortality in patients with peripheral arterial disease. J Hum Hypertens.

[12] Xiang Q, Li Y, Xia X, Deng C, Wu X, Hou L (2022). Associations of geriatric nutrition risk index and other nutritional risk-related indexes with sarcopenia presence and their value in sarcopenia diagnosis. BMC Geriatr.

[13] Abreo AP, Bailey SR, Abreo K (2021). Associations between calf, thigh, and arm circumference and cardiovascular and all-cause mortality in NHANES 1999-2004. Nutr Metab Cardiovasc Dis.

[14] Kanegusuku H, Cucato GG, Domiciano RM, Longano P, Puech-Leao P, Wolosker N (2020). Impact of obesity on walking capacity and cardiovascular parameters in patients with peripheral artery disease: A cross-sectional study. J Vasc Nurs.

[15] Barroso WK, Rodrigues CI, Bortolotto LA, Mota-Gomes MA, Brandão AA, Feitosa AD (2021). Diretrizes Brasileiras de Hipertensão Arterial - 2020. Arq Bras Cardiol.

[16] Siebenhofer A, Kemp C, Sutton A, Williams B (1999). The reproducibility of central aortic blood pressure measurements in healthy subjects using applanation tonometry and sphygmocardiography. J Hum Hypertens.

[17] Van Bortel LM, Duprez D, Starmans-Kool MJ, Safar ME, Giannattasio C, Cockcroft J (2002). Clinical applications of arterial stiffness, Task Force III: recommendations for user procedures. Am J Hypertens.

[18] Electrophysiology TF, Task Force of the European Society of Cardiology and the North American Society of Pacing and Electrophysiology (1996). Heart rate variability: standards of measurement, physiological interpretation and clinical use. Circulation.

[19] Gornik HL, Aronow HD, Goodney PP, Arya S, Brewster LP, Byrd L, Chandra V, Drachman DE, Eaves JM, Ehrman JK, Evans JN, Getchius TSD, Gutiérrez JA, Hawkins BM, Hess CN, Ho KJ, Jones WS, Kim ES, Kinlay S, Kirksey L, Kohlman-Trigoboff D, Long CA, Pollak AW, Sabri SS, Sadwin LB, Secemsky EA, Serhal M, Shishehbor MH, Treat-Jacobson D, Wilkins LR, Peer Review Committee Members (2024). 2024 ACC/AHA/AACVPR/APMA/ABC/SCAI/SVM/SVN/SVS/SIR/VESS Guideline for the Management of Lower Extremity Peripheral Artery Disease: A Report of the American College of Cardiology/American Heart Association Joint Committee on Clinical Practice Guidelines. Circulation.

[20] Ritti-Dias RM, Sant'anna FD, Braghieri HA, Wolosker N, Puech-Leao P, Lanza FC (2021). Expanding the use of six-minute walking test in patients with intermittent claudication. Ann Vasc Surg.

[21] McDermott MM, Ferrucci L, Gonzalez-Freire M, Kosmac K, Leeuwenburgh C, Peterson CA (2020). Skeletal muscle pathology in peripheral artery disease: a brief review. Arterioscler Thromb Vasc Biol.

[22] Rodríguez AJ, Karim MN, Srikanth V, Ebeling PR, Scott D (2017). Lower muscle tissue is associated with higher pulse wave velocity: A systematic review and meta-analysis of observational study data. Clin Exp Pharmacol Physiol.

[23] Tap L, Kirkham FA, Mattace-Raso F, Joly L, Rajkumar C, Benetos A (2020). Unraveling the links underlying arterial stiffness, bone demineralization, and muscle loss. Hypertension.

[24] Shirwany NA, Zou MH (2010). Arterial stiffness: a brief review. Acta Pharmacol Sin.

[25] Correia MA, de Sousa AS, Andrade-Lima A, Germano-Soares AH, Zerati AE, Puech-Leao P (2020). Functional and cardiovascular measurements in patients with peripheral artery disease: comparison between men and women. J Cardiopulm Rehabil Prev.

[26] McDermott MM, Ferrucci L, Liu K, Guralnik JM, Tian L, Kibbe M (2011). Women with peripheral arterial disease experience faster functional decline than men with peripheral arterial disease. J Am Coll Cardiol.

[27] Gardner AW, Parker DE, Montgomery PS, Khurana A, Ritti-Dias RM, Blevins SM (2010). Gender differences in daily ambulatory activity patterns in patients with intermittent claudication. J Vasc Surg.

[28] Abreo AP, Bailey SR, Abreo K (2021). Associations between calf, thigh, and arm circumference and cardiovascular and all-cause mortality in NHANES 1999-2004. Nutr Metab Cardiovasc Dis.

